# Application of steam explosion in oil extraction of camellia seed (*Camellia oleifera* Abel.) and evaluation of its physicochemical properties, fatty acid, and antioxidant activities

**DOI:** 10.1002/fsn3.924

**Published:** 2019-02-11

**Authors:** Shanying Zhang, Yong‐Gui Pan, Lili Zheng, Yang Yang, Xiaoyan Zheng, Binling Ai, Zhimin Xu, Zhanwu Sheng

**Affiliations:** ^1^ Haikou Experimental Station Chinese Academy of Tropical Agricultural Sciences Haikou China; ^2^ College of Food Science Hainan University Haikou China; ^3^ School of Nutrition and Food Science Louisiana State University Agricultural Center Baton Rouge Louisiana

**Keywords:** camellia seed oil, physicochemical properties, steam explosion, volatile compounds

## Abstract

This study evaluated the physicochemical properties of oils extracted from steam‐exploded camellia seed (*Camellia oleifera* Abel.). Steam pressure, resident time, fatty acid composition, total phenolics, tocopherol, squalene, and sterol contents, and volatile compounds were determined. ^1^H NMR and FTIR spectra were performed for the structure of camellia seed oil. This study has found the highest yield of oil was 86.56% and was obtained when steam explosion pretreatment was at 1.6 MPa 30 s. Oil extracted by steam explosion pretreatment exhibited favorable physicochemical properties and stronger antioxidant activities compared to untreated oil. The compositions of fatty acid were similar between treated and untreated camellia seed oil. According to the ^1^H NMR and FTIR analyses, the functional groups of the oils were not significantly affected by the steam explosion pretreatment. Furans such as 2‐pentyl‐furan, 2‐furanmethanol, and 3‐methyl‐furan were produced from stream‐exploded camellia seed. Scanning electron microscopy revealed that steam explosion pretreatment efficiently promoted the release of oil by destroying the cell structure of camellia seed. Therefore, steam explosion can be an effective method for the camellia seed oil extraction.

## 1 INTRODUCTION

Camellia oleifera (*Camellia oleifera* Abel.) is a member of the *Theaceae* family and the most important edible oil tree growing specifically in China (Xiao et al., 2016). Camellia seed oil is used extensively in China as cooking oil, and it mainly distributed and cultivated in southern China. *Camellia* has been produced for more than 1,000 years, and the annual output of camellia seeds exceeds 250 million kg (Fang, Fei, Sun, & Jin, 2016). The oil quality has been proved to be better than peanut, rape, palm, and bean oil, and could even surpass olive oil (Zhang, Wang, Wu, Xu, & Chen, 2011), because camellia seed oil is rich in unsaturated fatty acids, as well as vitamins and minerals, and other bioactive components (Ma, Ye, Rui, Chen, & Zhang, 2011). For example, the oleic acid content of camellia seed oil was 77.93%, while that of olive oil was 76.16% (Wang, Zeng, Verardo, & Contreras, 2017). In addition, *camellia* oil also contains many healthy and beneficial bioactive compounds such as sterols, squalene, and tocopherols (Xiao et al., 2017), which have been characterized as having health‐promoting effects include lowering blood pressure, atherosclerosis, and scavenging free radicals (Lee & Yen, 2006). It also exerts potent antiulcer effects against oxidative damage in the stomach and intestine induced by ketoprofen (Cheng et al., 2014). Also, *camellia*a seed oil could be a valuable raw material and functional product to the food industry. For example, *camellia* oil is used as a natural antioxidant to improve the stability of many oil, and it effectively delays the development of pericarp browning and the loss of red color in litchi fruit because it contains high levels of antioxidants and vitamins such as phenolics (Zhang et al., 2017).

Oil extraction from *Camellia oleifera* seed is a difficult process and *camellia* seed contains tea saponin, which is a foam‐stabilizing and excellent emulsifying agent (Zhang, Han, et al., 2012; Zhang, Zhang, & Chen, 2012). Because of this, the difficulties of separating oils have made it challenging to provide an efficient yield. Until now, many techniques such as organic solvent extraction (Lee & Yen, 2006), subcritical water extraction (Wu et al., 2018), ultrasound‐assisted (Wu & Li, 2011), microwave puffing‐pretreated (Zhang & Jin, 2011), microwave puffing and aqueous enzymatic extraction (Zhang, Han, et al., 2012; Zhang, Zhang, & Chen, 2012), and aqueous enzymatic process (Fang et al., 2016) have been employed to extract oil from camellia seed. However, among them are used of chemicals (such as, Petroleum ethe and hexane) which can lead to environmental pollution and may require additional processes to remove or neutralize the chemicals used. Aqueous enzymatic extraction is a promising method and has been developed and performed in the laboratory which can improve the free fatty acid, vitamin E, and squalene contents, and physicochemical properties of oil (Fang et al., 2016). However, aqueous enzymatic method comes at a high cost. Another reason is that the aqueous enzymatic method cannot produce the desired aroma of oil. Consequently, searching for an efficient technique with low cost and high extraction of oil is vitally important for the extensive utilization of *camellia* seed.

Steam explosion is an innovational, economical, and effective method, which is explored extensively used for the pretreatment of cellulose (Deepa et al., 2011), hemicellulose (Jiang et al., 2017), and lignin (Chang et al., 2012). The steam explosion has also been used to extract protein (Zhang, Yang, Zhao, Xiao, & Zhang, 2013), flavonoids (Song et al., 2014), polyphenols (Chen & Chen, 2011a), antioxidant compounds (Gong, Huang, & Zhang, 2012), and ethanol (Rocha, Gonçalves, Oliveira, Olivares, & Rossell, 2012). The principle of the treatment is the use of steam hydrolysis at high temperature and pressure, followed by sudden decomposition of the materials biomass with low molecular weight substance produced. In a steam explosion process, high saturated steam pressure is rapidly released to ambient, in 0.00875 s; meanwhile, high temperature decreased quickly and cooling the materials in the process. This is different from other normal thermal pretreatment (Gong et al., 2012). Compared with other pretreatments, the advantages of steam explosion include a significantly lower energy cost and environmental friendly (Zhang et al., 2013). In recent years, this technology has been used for the extraction process in studies regarding oil extraction, such as that of Ni, Zhao, Zhang, Gasmalla, and Yang (2016) which reported that steam explosion is a highly effective method for extracting oil from corn germ. And used steam explosion to extract oil from sumac fruit, their results showed that the oil yield at equilibrium increased to 16.04%, approximately fourfold higher than that of the raw sample (Chen & Chen, 2011b). Also, the oil extraction yield was improved, and the mass transfer coefficient according to the kinetics of the oil extraction of sesame seed was decreased in sesame seed (*Sesamum indicum* L.). In other words, steam explosion enhanced the oil extraction efficiency and it has proven to be an efficient method for releasing oil from vegetable seed.

However, using steam explosion in oilseeds for enhancing free oil yield is exceedingly limited, the application of steam explosion pretreatment for camellia seed, to the best of our knowledge, has not yet been reported. Therefore, the fatty acid compositions, physicochemical properties, total phenolic, total tocopherols, squalene, and sterol contents are evaluated and determined using ^1^H NMR and FTIR. In addition, the microscopic structures of material before and after steam explosion are observed to clarify extraction mechanisms.

## 2 MATERIALS AND METHODS

### 2.1 Materials and reagents

The camellia seeds (*Camellia oleifera* Abel.) were provided by a local forest farm (Haikou country, Hainan province, China). The raw material (dry camellia seeds) contained 9.38% moisture content. The seeds were sealed in plastic containers and stored in a refrigerator at 4°C until extraction.

Fatty acid standards, gallic acid, Folin phenol reagent, α‐tocopherol, squalene, 5α‐cholestane, and 2,2‐diphenyl‐1‐picrylhydrazyl (DPPH) were purchased from Sigma‐Aldrich Co. (Steinheim, Germany). All other reagents and chemicals were of analytical grade and purchased from Guangzhou Chemical Reagents Co (Guangzhou China).

### 2.2 Steam explosion and oil extraction

All steam explosion experiments were carried out in the QBS‐80B SE device with a 0.4 L chamber from Gentle Bioenergy, Henan, China. 300 g of camellia seeds was placed inside the vessel and exposed to the saturated steam. The steam pressures were set at 0–2.3 MPa. The resident time was performed in the range of 0–120 s, and finally termination by explosive decompression. The exploded materials were collected and dried in a ventilated drying oven for 5 hr at 50°C.

The dried camellia seeds were ground in a high‐speed medicine grinder (Y‐800, Kemanshi, China). The ground camellia seeds were then coarsely grounded, after passing through a set of standard‐mesh sieves (40 mesh), the milled camellia seeds were stored in 4°C fresh keeping cabinet until used for the extraction experiments.

Camellia seed oil was extracted from milled camellia seed using an aqueous as solvent. For each extraction, 5 g of camellia seed powder and extraction solvent of specified volume (solid‐liquid ratio was set as 1:4.5 [w/v, kg/L]) were added into a flask, the pH value of system was adjusted to 9.0 using 1 M NaOH and 0.5 M HCI solution, and shaking horizontally was done for 2.5 hr at 75°C in a rotary shaker. The mixture was centrifuging at 6986 *g* for 10 min, then it was kept frozen at −20°C for 12 hr and thawed at room temperature. After demulsification, the slurry was centrifuging at 4,000 rpm for 10 min. The oil content of the entire fruit and its isolated parts was determined by Soxhlet extraction using petroleum ether.

Three replicates of the experiments were conducted. The amount of extracted oil was calculated gravimetrically after collection, and the free oil yield is expressed as follows:

$$\text{Extraction oil of yeild}\;\left(\%\right)=\left(\frac{\mathrm{mass}\;\mathrm{of}\;\mathrm{extracted}\;\mathrm{oil}}{\mathrm{mass}\;\mathrm{of}\;\mathrm{Soxhlet}\;\mathrm{extracted}\;\mathrm{oil}}\right)\times 100$$

### 2.3 Physicochemical properties

The acid, peroxide, iodine, and saponification values were determined according to standard methods of American Oil Chemist's Society (AOCS, 2009).

### 2.4 Determination of the fatty acid compositions

To analyze the fatty acid composition of camellia seed oil, the oil (60 mg) was firstly converted into fatty acid methyl esters (FAME) using 2 ml NaOMe (0.5 M). The mixture was bathed in water for 30 min at 65°C, then 2 ml methanolic boron trifluoride (15%) was added in the mixture, continuously bathing for 5 min. Lastly, 1 ml saturated sodium chloride solution and 4 ml n‐hexane were added immediately followed by vigorous shaking for 30 s. The anhydrous sodium sulfate was added; after stratification, the upper isooctane layer (1 ml) was filtered at 0.45 μm.

The fatty acid composition of oil was determined with a gas chromatograph (Agilent 7890B) equipped with a FID and a HP‐5 column (30 m, 0.32 mm i.d., 0.25 μm film thickness; Supelco, USA). The nitrogen was used as a carrier gas at a flow rate of 1 ml/min. Sample was injected (1 μl) with a split mode (ratio 10:1). Injector temperature and detector temperature were set at 250 and 300°C, respectively. Oven temperature increased from 100°C (1 min) to 190°C at a rate of 5°C/min and was further increased at a rate of 1°C/min to a final temperature of 220°C. Fatty acids were identificated with retention times obtained from commercial FAME standards (Sigma Chemical, St. Louis, MO). All experiments were carried out in triplicate sets. The relative amount of each fatty acid was calculated from the integrated area of each peak and expressed as a percentage of the total area of all peaks.

### 2.5 Total tocopherols (TT) and total phenolics (TP) contents

The TP content of camellia seed oil was extracted by methanol–water solution (80%:20% v/v) and determined by Folin–Ciocalteu method according to the colorimetric method described previously by Delfan‐Hosseini, Nayebzadeh, Mirmoghtadaie, Kavosi, and Hosseini (2017). A calibration curve of gallic acid in methanol was carried out in the concentration ranges of 0.04–0.40 mg/ml. The results were expressed as μg gallic acid equivalent per gram of oil samples. Triplicate test was performed for each sample.

Tocopherols content of the extracted oils was determined using UPLC method with fluorescence detection. Waters liquid chromatography system equipped with a column heater, a photodiode array detector ACQ‐FLR, controlled by Waters Empower chromatographic software. In all analyses, an Acquity UPLC Waters BEH C18 column of 1.7 μm (2.1 × 50 mm) was used. The analysis was carried out at 35°C temperature under isothermal condition, and the mobile phase was composed of 100% acetonitrile. The volume of injection is 10 μl, and the flow rate was 0.5 ml/min. Using FLR to detect and quantify α‐tocopherol, the excitation wavelengths and emission wavelengths are 294 nm and 338 nm, respectively. A calibration curve of α‐tocopherol in toluene was performed in the concentration ranges of 0–300 mg/ml. Results were expressed in mg of α‐tocopherol per kilogram of oil.

### 2.6 Determination of squalene and sterol contents

The content and composition of the sterols and squalene were determined by gas chromatography (GC) following procedure reported by Wang et al. (2017) with some modification. Camellia seed oil (1.5 g) was saponified with 50 ml of 1 M methanolic potassium hydroxide at 85°C for 1 hr in a reflux condenser. After cooling, 50 ml of saturated sodium chloride was added, and the unsaponifiable matter was extracted with 3 × 50 ml of n‐hexane. The combined n‐hexane fractions were washed 3–5 times with distilled water (30 ml). Anhydrous sodium sulfate was added to the n‐hexane layer (in order to eliminate aqueous residues), the organic layer was then evaporated at 40°C on a rotary evaporator, and the residue was redissolved in 5 ml of n‐hexane. Finally, the extract was filtered through a 0.45 μm syringe filter and stored at −20°C until analysis.

The samples were analyzed on a GC (Agilent 7890B) equipped with a FID and a HP‐5 column (30 m × 0.32 mm × 0.25 μm; Supelco, USA). Helium was used as the carrier gas at a flow rate of 1 ml/min. A sample of 1.0 μl was injected in a splitless mode with an injector temperature of 250°C. The column temperature was held at 100°C for 5 min, then increased to 180°C at a rate of 25°C/min, held for 1 min, then further increased at a rate of 10°C/min to a final temperature of 280°C. The detector temperature was set at 300°C.

According to the retention times of reference samples of sterols (5α‐cholestane) and squalene, results were expressed as mg/100 g of oil. All samples were analyzed in triplicate and means of the results are reported.

### 2.7 Antioxidant activity evaluation

The seed oil obtained under the optimum conditions was subjected to screening for its possible antioxidant activity. The antioxidant activity was assessed using 1,1‐diphenyl‐2‐picrylhydrazyl (DPPH) radical‐scavenging assay was performed as described by Delfan‐Hosseini et al. (2017) with some modifications. An aliquot of seed oil (100 μl) was mixed with 3.9 ml of methanol DPPH solution (0.2 mM), then incubated in the dark for 60 min at room temperature. All the data were the averages of triplicate determinations of three independent tests. The absorbance of the solutions was measured at 517 nm using a UV‐Vis spectrophotometer (UV‐1800). The results were expressed by IC_50_ values which corresponded to the concentration of oil (mg/ml) neutralizing 50% of DPPH radicals.

### 2.8 Scanning electron micrographs (SEM) observation

In order to investigate the influence of steam explosion, microstructure observations of raw and the optimum conditions camellia seed oil were carried out via SEM (JEOL JSM‐7500F, Tokyo, Japan). Samples were dried, fixed, and coated by gold, and then examined under high vacuum condition at an accelerating voltage of 10.0 kV (20 μm, 1,800 magnification).

### 2.9 Acquiring ^1^H NMR spectra analysis

^1^H NMR spectroscopy was used to obtain maximum possible information on positional distribution of fatty acids in camellia oil. 15–25 mg of sample was dissolved in a mixture of 0.5 ml of CDCl_3_. These mixtures were thoroughly mixed well and then transferred to NMR tubes for ^1^H NMR spectra analysis. The spectra were recorded at 25°C on a Bruker Av500 NMR spectrometer (Bruker BioSpin GmbH, Germany, ^1^H frequency 500.13 MHz) equipped with inverse detection (^1^H–^13^C–^15^N) system. The resulting spectra were processed using Bruker Topspin 3.5 software (Bruker Biospin, Rheinstetten, Germany). The data were processed without zero‐filling and by using exponential multiplication with a line‐broadening of 1.0 Hz.

### 2.10 Acquiring Fourier transform infrared (FTIR) spectra

A FTIR spectrophotometer (AVATAR 370 FIR, Thermo Nicolet) was utilized to record the percent transmittance in the absorption mode 400 to 4,000 cm^−1^ at a resolution of 4 cm^−1^. A small amount (3–5 μl) of the extracted oil sample was deposited between the two well‐polished KBr pellet and the Pasteur pipette was used to create a thin film.

### 2.11 Identification of volatile compounds

Volatile compounds were determined using a GC (Agilent 7890B, Heilbronn, Germany) coupled to a mass spectrometer (Agilent 5974, Heilbronn, Germany) and a Headspace sampler (Agilent 7697A, Heilbronn, Germany). 10.00 g of camellia oil was introduced into 20‐ml headspace vials of headspace analyzer. The vials were sealed air‐tight with a silicone/polytetrafluoroethylene (PTFE) septum. Samples were subjected to dynamic headspace for 30 min at 200°C.

Chromatographic separation was performed on an HP‐5 column (30 m × 0.32 mm × 0.25 μm; Supelco, USA). Helium (purity 99.99%) was used as a carrier gas at a constant pressure of 16 psi. Samples were injected in a splitless mode. The temperature program was as follows: 3 min at 40°C, first ramp 5°C/min to 250°C, and total analysis run was 60 min. The mass spectrometer was operated in electron impact (EI) ionization mode at 70 eV using full‐scan mode from m/z 25 to 550. Source and quadrupole temperatures were 230 and 150°C, respectively.

### 2.12 Statistical analyses

All experimental measurements were conducted at least in triplicate and data are expressed as mean ± standard deviation, where feasible. The data obtained in this study were analyzed by one‐way analysis of variance (ANOVA) using SPSS 16 (SPSS Inc., Chicago, IL). Statistical significance was considered at the 5% level (*p* < 0.05).

## 3 RESULTS AND DISCUSSION

### 3.1 Effect of steam explosion conditions on oil extraction

**Figure 1:**
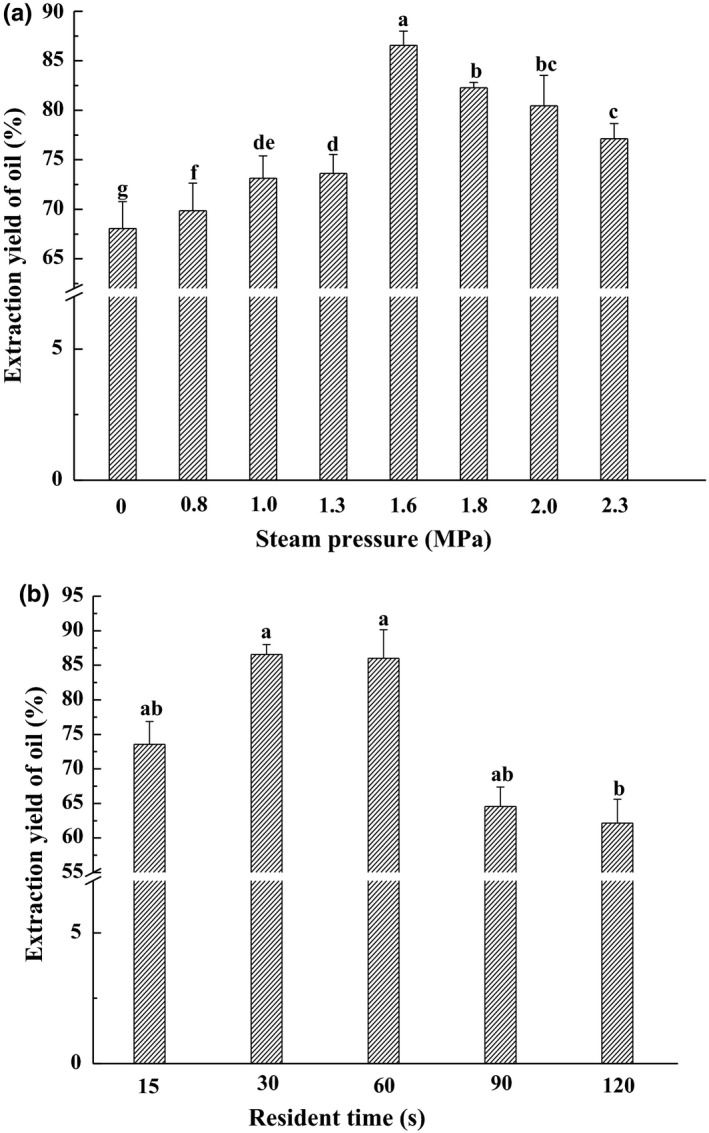
Effect of steam explosion on extraction yield of oil, residence time was kept constant for 30 s (a); effect of residence time, steam pressure was kept constant for 1.6 MPa (b). Bars (mean ± *SD*,* n* = 3) with different letters have mean values that are significantly different (*p* < 0.05)

The effect of steam explosion at 30 s for different pressure on camellia seed oil extraction is shown in Figure 1a. Steam explosion treatment had the highest oil yield of 86.56% at 1.6 MPa from the yield of 68.06% for untreated sample. Simultaneously, free oil content had gradually improved with the increase of pressure from 0 to 1.6 MPa, this is due to the fact that in some pressure range, steam explosion could destroy the cell thoroughly and enhance the porosity, which released the oil surrounded in the cell and decreased the oil in the seed cake. However, after 1.6 MPa, the yield dropped slightly for higher steam pressure. Resident time was another main parameter of steam explosion which has a greater impact on the oil yield. In Figure 1b, the free oil yield peaks was showed at 30 s. However, there is no significant difference between 30 and 60 s, but free oil yield at other resident time was significantly lower than 30 s. That is because higher pressure and resident time may urge protein denaturation and aggregation (Ni et al., 2016). Oil droplets were wrapped in aggregated protein and are difficult to release. However, higher pressure and resident time could improve the content of tea saponin—one of the emulsifiers, which would increase emulsification and prevent the oil from the emulsion after centrifugation (Fang et al., 2016). In addition, the color of oil from steam‐exploded camellia seed was slightly darker than that of the untreated camellia seed oil. The probable cause is most likely due to the Maillard reaction. Therefore, appropriate steam pressure and resident time could strengthen the performance of steam explosion treatment, by destroying the cell wall and disconnecting the body to increase oil extraction as well as excessive pressure or time would result in a decrease the yield of free oil. Thus, in consideration of oil recovery and time‐consumption, 1.6 MPa 30 s was taken as the optimal steam explosion conditions of treating camellia seed.

### 3.2 Physicochemical properties

**Figure 2:**
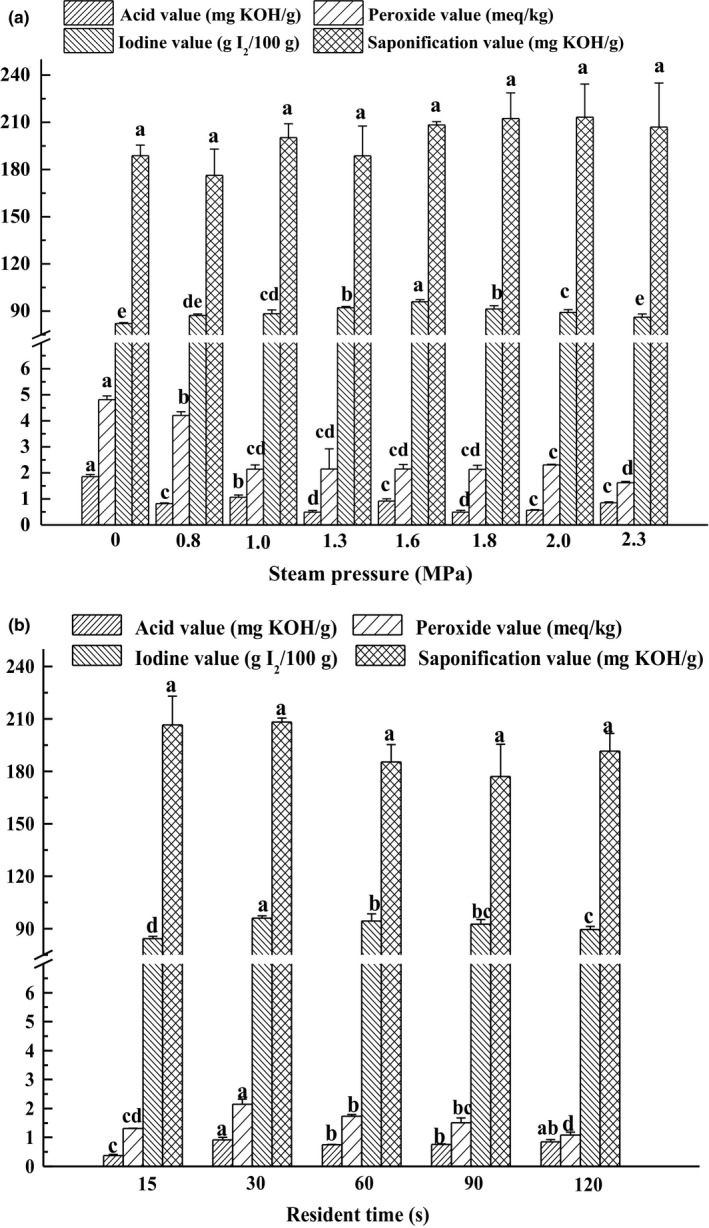
Effect of steam explosion on physicochemical properties of extraction oil, residence time was kept constant for 30s (a); effect of residence time, steam pressure was kept constant for 1.6 MPa (b). Bars (mean ± *SD*,* n* = 3) with different letters have mean values that are significantly different (*p* < 0.05)

To understand the effect of steam explosion on the physicochemical properties of camellia oil, the acid, peroxide, iodine, and saponification value were analyzed, which are shown in Figure 2a,b. The oil samples used for analysis were unrefined oils. Corresponding to 0–2.3 MPa employed in steam pressure and 0–120 s applied in resident time might affect the physicochemical characteristics of the extracted oil.

As shown in Figure 2a, acidity decreased with different steam pressure pretreatment, and all treated sample's acidity was lower than raw materials (1.86 ± 0.08 mg KOH/g). The changes of peroxide value also show a similar trend, and all steam‐exploded sample's peroxide value was lower than raw materials (4.81 ± 0.14 meq/kg). As for different resident time (Figure 2b), both acid and peroxide value showed a peak at 1.6 MPa 30 s, 0.92 ± 0.09 mg KOH/g and 2.15 ± 0.17 meq/kg, respectively, still lower than untreated seed oil. In theory, oil extracted from pretreated oilseeds at high temperatures is prone to hydrolysis, which will lead to higher acid value than that of raw sample. The lower acidity of the camellia oil in the steam‐exploded sample may be due to the short retention time and evaporation of free fatty acids in the oil at the moment of explosion. Acid value of steam‐exploded sumac fruit also decreased with corresponding pressure 1.3–1.5 MPa (Chen & Chen, 2011b). And, Timilsena, Vongsvivut, Adhikari, and Adhikari (2017) also reported that the low acid and peroxide value indicated that chia seed oil embodied lower quantities of oxidation by‐products and free fatty acids including hydroperoxides and aldehydes. Oil sample of low acid value and peroxide value can be regarded as good quality.

The effect of different steam pressure on iodine value of camellia is presented in Figure 2a. The iodine value of steam‐exploded samples increased with the increase of pressure from 0 MPa to 1.6 MPa, after which, it decreased to 2.3 MPa. Also, as for different resident time which showed in Figure 2b, the iodine value showed a peak at 30 s (95.97 ± 1.37 g I_2_/100 g) higher than other resident time. All steam explosion treated samples extracted an oil higher in iodine value compared with untreated samples (82.13 ± 0.57 g I_2_/100 g), indicating a higher degree of unsaturation fatty acids (Jiao et al., 2014), which agreed with a high oleic acid content (Zhang & Jin, 2011).

The saponification values indicated short‐chain fatty acids levels in vegetable oils (Timilsena et al., 2017). As illustrated in Figure 2a,b, the saponification values of the camellia seed oils ranged from 176.32 ± 16.62 to 213.29 ± 21.01 mg KOH/g oil, however, had no significant difference was found between the saponification value and steam pressure or resident time, which showed that steam explosion has no significant influence on the levels of short‐chain fatty acids.

### 3.3 Total tocopherols, total phenolics, squalene, and sterol contents

**Figure 3:**
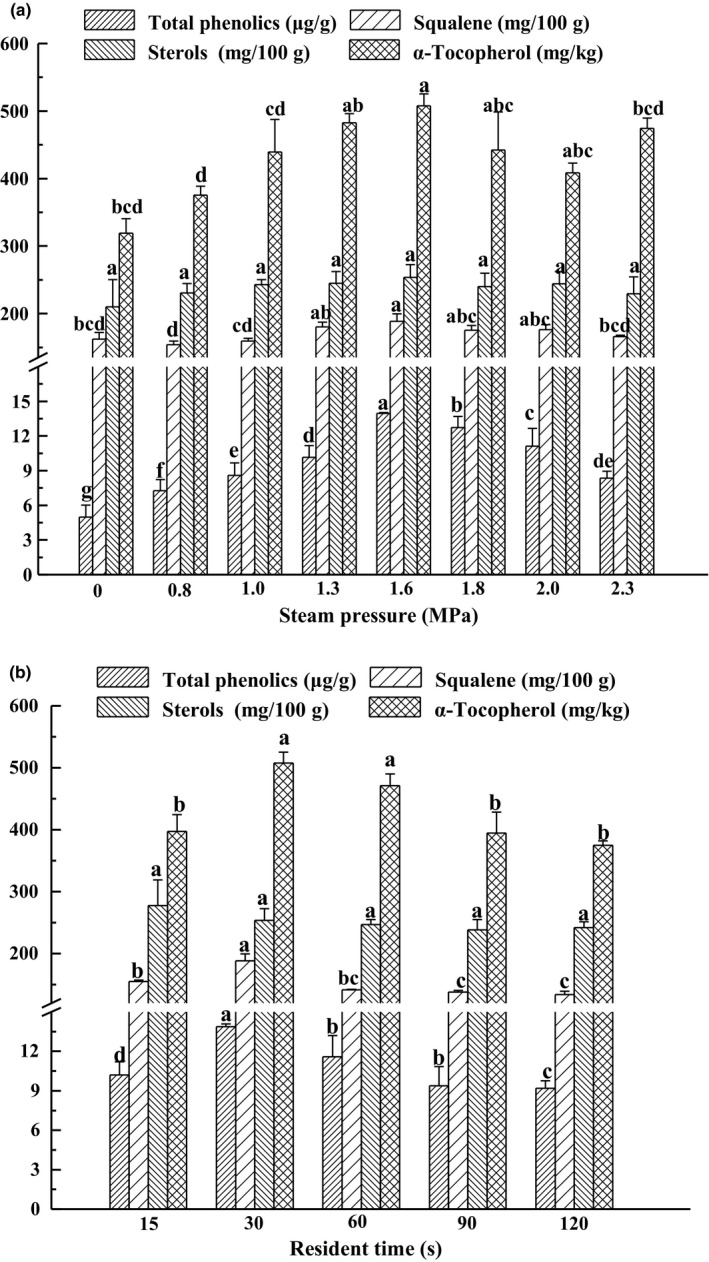
Effect of steam explosion on bioactive compound of extraction oil, residence time was kept constant for 30 s (a); effect of residence time, steam pressure was kept constant for 1.6 MPa (b). Bars (mean ± *SD*,* n* = 3) with different letters have mean values that are significantly different (*p* < 0.05)

Tocopherols and phenolics are considered to be the best‐known natural antioxidants in plants and they are thought to break the main chain of antioxidants in the free radical chain reaction or convert the lipid radicals into more stable products, which have an important role in improving the antioxidant capacity of vegetable oils (Jiao et al., 2014). Squalene and sterols are also biologically active compounds that are very closely related to the quality of the oil, and they can form a major proportion of the unsaponifiables (Azadmard Damirchi & Dutta, 2006). For example, the physiologic functions of squalene and sterols include antioxidant, anticancer, reducing cholesterol and triglyceride levels in serum (Salvo et al., 2017; Sodeif, Fatemeh, Javad, Mahbob, & Bahramfathi, 2010; Xiao et al., 2016). As illustrated in Figure 3a, total phenolics, α‐tocopherol, squalene and sterol, the amount of these four bioactive compounds in oil showed similar trends, all of them were present in the highest amount (13.96 ± 0.08 μg GAE/g oil, 507.85 ± 17.62 mg/kg, 188.34 ± 11.46 mg/100 g and 253.52 ± 19.02 mg/100 g) at 1.6 MPa 30 s. The amount of these four bioactive compounds in seed oil treated with other steam explosion pressures are less than 1.6 MPa 30 s, but higher than that of without steam explosion pretreatment extraction (5.00 ± 1.02 μg GAE/g oil, 319.08 ± 21.59 mg/kg, 162.38 ± 9.67 mg/kg and 186.69 ± 40.10 mg/100 g). As shown in Figure 3b, the content of total phenolics, α‐tocopherol, and squalene at 30 s was higher than that of other resident time treatments. Although the amount of sterols showed a peak at 15 s, there is no significant (*p* < 0.5) difference between 15 s and 30 s for all the content of them higher than untreated seed. These results may explain the fact that steam explosion pretreatment destroyed the intact cellular structure of oilseed, increased the release of α‐tocopherols, total phenolics, squalene, and sterols in short time, thereby enhancing the bioactive components in the extracted oil as shown in Figure 3a,b. However, if the resident time of steam explosion is too long or the steam pressure is too high, steam explosion pretreatment will reduce the α‐tocopherols, total phenolics, squalene, and sterol contents of extracted oils. This may be due to the fact that bioactive compounds are easily decomposed to some extent by either relatively long time or exposure too extreme high pressure saturated steam and temperature.

### 3.4 Fatty acid composition of oils

The percentages of the fatty acids of oil from camellia seed were presented in Table 1. It can be observed that the tested oils contained seven common fatty acids. Among them, oleic (18:1), palmitic (16:0), linoleic acid (18:2), and α‐linolenic acid (18:3) were the four principal compounds, which accounted for 98.0%–99.0% of the total fatty acids profiles, and oleic acid being of monounsaturated fatty acids (MUFA) was the most abundant in steam explosion and untreated camellia seed oil. The contents were 81.05 ± 0.08 (1.6 MPa 30 s) and 79.16 ± 0.69 (0 MPa 30 s), respectively, and the pretreatment of oils by different methods was significantly different. These findings were also in agreement with the results of iodine value. The seven main fatty acids detected in camellia seed oils were different from the reported results in others’ studies. For example, Zhang and Jin (2011) used solvent extraction in conjunction with the microwave puffing pretreatment on the camellia seed, eleven fatty acids were analyzed by GC/MS, and the oleic acid content was 75.05 ± 0.15%, which were lower than those in untreated and steam‐exploded seed. Wang et al. (2017) also found that compared with Oleifera and Camellia oils, there was a significant difference in the content of oleic acid, linoleic acid, and palmitic acid in thea oil. These dissimilarities could be ascribed to different causes, such as genotype, growing condition, method of extraction (Huang, Wang, & Liang, 2015). In addition, the refining conditions for camellia oil, such as different temperatures, can also have a great influence on the type and content of camellia seed fatty acids (Wei et al., 2015).

**Table 1:** Fatty acid composition (%) of camellia seed oil obtained from steam explosion and untreated

NO	Fatty acid	0 MPa	1.6 MPa 30 s
1	Myristic acid (14:0)	0.05 ± 0.004	0.04 ± 0.004^a^
2	Palmitic acid (16:0)	11.09 ± 0.10^a^	10.23 ± 0.09^b^
3	Stearic acid(18:0)	1.08 ± 0.01^a^	1.07 ± 0.005^b^
4	Oleic acid (18:1)	79.16 ± 0.69^b^	81. 05 ± 0.08^a^
5	Linoleic acid (18:2)	5.52 ± 0.68^a^	4.96 ± 9.09^b^
6	α‐linolenic acid (18:3)	2.67 ± 0.11^a^	2.66 ± 0.08^a^
7	Peanut acid (20:0)	0.42 ± 0.006^a^	0.46 ± 0.004^a^
Total	Saturated fatty acids	12.65	12.40
Total	Monounsaturated fatty acids	79.16	81.05
Total	Polyunsaturated fatty acids	8.19	8.04

Different small letters (a, b) within a row are significantly different at *p* < 0.05.

### 3.5 Antioxidant activity

Antioxidants could scavenge the free radicals of oil and inhibit the chain reaction and thus prevent the oxidation of lipid (Samaram et al., 2015). The antioxidant activities of camellia seed oils pretreatment with steam explosion or not were assessed using DPPH radical‐scavenging assay. Oil with steam explosion pretreatment (1.6 MPa 30 s) exhibited significantly (*p* < 0.05) superior efficacy in scavenging the DPPH radicals (24.65 ± 1.23 mg/ml) compared with untreated oils (29.74 ± 1.09 mg/ml). A variety of bioactive components of camellia seed oil have the major effect on its oxidative stability behavior, such as tocopherols, total phenolics, squalene, and sterols (Ma et al., 2011). And other study has shown that the composition of fatty acids plays a vital role in the antioxidant activity of oil, especially the high level of unsaturated fatty acids (Hu et al., 2017). The antioxidant activities of camellia seed oils were improved mainly because steam explosion pretreatment enhances the content of tocopherols, total phenolics, squalene, sterols, and unsaturated fatty acid, resulting in a much higher availability of such bioactive components into oils. These results were in agreement that there was a positive correlation between the content of these bioactive components and antioxidant activity of oils. Methanol extract of tea seed oil showed higher antioxidant capacity by improving the content of total phenolics, but the transparency, odor, and flavor of solvent extracted oil were not satisfactory (Long et al., 2012).

### 3.6 Analysis of microscopic changes

**Figure 4:**
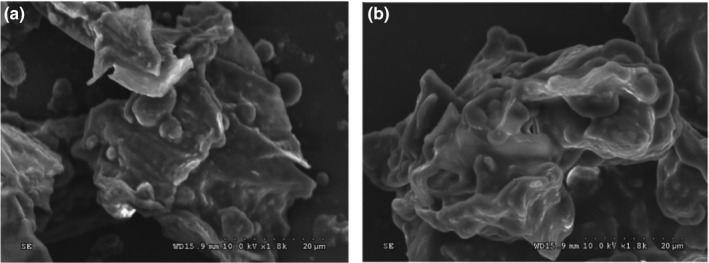
Scanning electron micrographs of camellia seed: camellia seed without treatment (a); camellia seed treated with steam explosion (b)

To gain further insight into the effect of the steam explosion pretreatment on the oil extraction from camellia seed and to understand the extraction mechanism, the camellia seed powder was examined by SEM to elucidate the morphological changes of steam‐exploded and raw camellia seed. The obvious effect of steam explosion pretreatment on the morphology of camellia seed could have been observed in Figure 4. Untreated camellia seed still possesses relatively complete structures, regular or compact shapes, and smooth surfaces, which is not conducive to the release of oil from the seed (Figure 4a). On contrary, steam‐exploded seed surface became rough and fissured and the morphology of samples was obviously broken down (Figure 4b). During steam explosion process, high temperature and pressure could cause physicochemical modifications structural, resulting in fissures and cavities, which in agreement with the previous study (Chen & Chen, 2011a,b). As a result of the reduced particle size, mass transfer resistance, and the generation of micropores, surface area increases giving the high oil yield. This result was consistent with the above‐mentioned high oil yields in the extraction process of oil from steam‐exploded camellia seed also performed steam explosion pretreatment which helped increase sesame seed oil yield at equilibrium. The SEM results indicated that the steam explosion technique efficiently promoted the release of oil by breaking down the cell structure of camellia seed. Until now, no study on the cell structures of steam explosion pretreatment camellia oil seed samples has been reported.

### 3.7 ^1^H NMR spectroscopy

**Figure 5:**
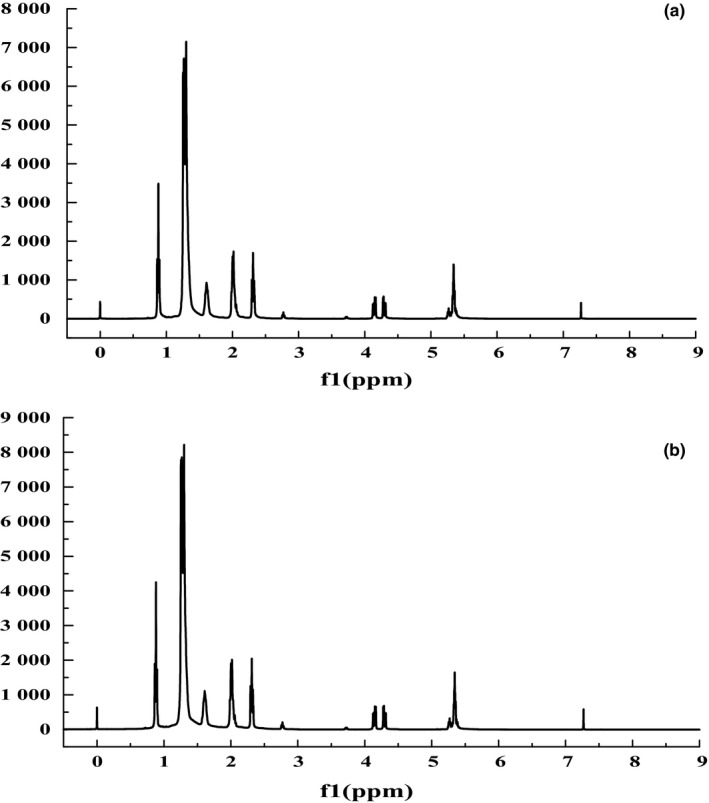
The ^1^H NMR spectrum of the extracted oils from untreated camellia seed (a); steam‐exploded camellia seed (b)

The ^1^H NMR spectra of the extracted oil from steam explosion and untreated camellia seed are presented in Figure 5. The majority of the characteristic peaks were observed in the range of 3.0–0.5 ppm. The region between 0.5 and 5.5 ppm in both oils contains all the typical ^1^H NMR signals of vegetable oils. The peak at 0.86 ppm and 0.95 ppm was assigned to terminal‐CH_3_. The resonances for ‐(CH_2_)n‐ (acyl chains) were identified at 1.22–1.30 ppm. The peaks at 1.61 ppm were due to β‐carbonyl methylene protons, while those found at 2.29–2.34 ppm were related to α‐methylene protons (Sherahi, Shahidi, Yazdi, & Hashemi, 2017). A triplet centered around 2.25–2.79 ppm due to methylene protons in the carbonyl. The peaks at 4.12–4.32 ppm were related to the hydrogen atoms on 1 and 3 carbon atoms of the glyceryl methylenes. The multiples at 5.27–5.37 ppm were attributed to olefinic hydrogen atoms of the different acyl groups. Otherwise, the peak at 7.26 ppm was attributed to CDCl_3_.

As it can be observed from Figure 5, steam explosion pretreatment could not change the peak time of the ^1^H NMR of the hydrogen. This result indicates that steam explosion did not change the type of fatty acids in the camellia oil. And the intensity of the signal related to linoleic acyl groups in the original sample (0 Ma 30 s) was less than that obtained for the steam explosion sample (1.6 MPa 30 s). Moreover, the intensity of this signal of the treated was higher that of untreated sample, indicating that the steam‐exploded sample has a higher oxidation stability than the untreated oil. This result is consistent with the results observed in the DPPH analyses.

### 3.8 FTIR spectroscopy

**Figure 6:**
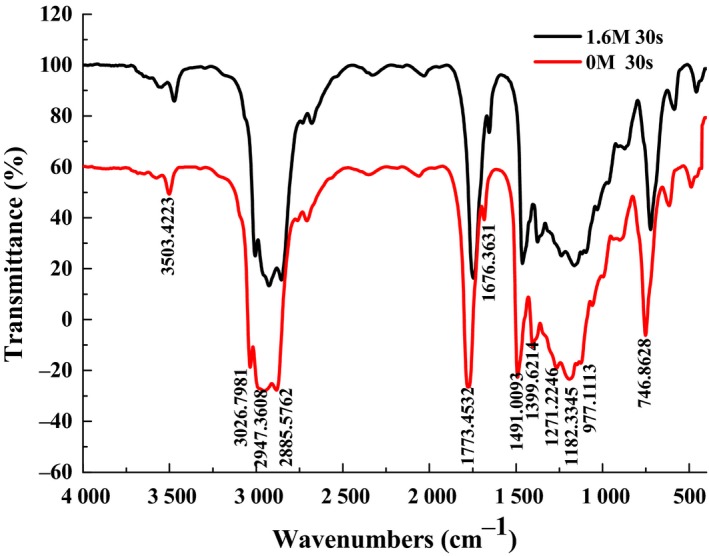
The ^1^H NMR spectrum of the extracted oils from untreated camellia seed (a); steam‐exploded camellia seed (b)

FTIR spectroscopy can be used to monitor structure changes in oils and fats and reveal the characteristic peak that specifically represents unsaturated fatty acids (UFAS) in the observed spectrum. To investigate whether steam pressure pretreatment produces structural changes to camellia seed oil, FTIR analysis was performed. The representative FTIR spectra of steam explosion and untreated camellia seed oil are presented in Figure 6. In general, the small band at 3,503 cm^−1^ is due to the overtone of the glyceride ester carbonyl absorption. The peak at 3,026 cm^−1^ can be attributed to the stretching vibration of cis lefinic CH double bands. The vibration at 2,947 and 2,885 cm^−1^ correspond to the methylene asymmetrical and symmetrical vibrations, respectively. The stretching vibration of the C=O group of triacylglycerols while that at 1,773 cm^−1^ was assigned, being characteristic of oils with a high unsaturation degree. The weak band at 1,676 cm^−1^ corresponds to the di‐substituted cis C=C of the unsaturated acyl groups, while the band at 1,491 cm^−1^ was due to the bending vibrations of the CH_2_ and CH_3_ aliphatic groups. The peaks at 1,271 cm^−1^ were related to the hydrogen atoms on 1 and 3 carbon atoms of the glyceryl methylenes. The absorption band at 1,182 cm^−1^, typical of plant seed oil, was attributed to the stretching vibration of the C‐O ester groups and the bending vibration of the CH_2 _group. The band at 977 cm^−1^ is assigned to the C‐O group in esters, and at 746 cm^−1^ originated from the =C–H bending out‐of‐plane for alkenes (Gutiérrez, Quiñones‐Segura, Sanchez‐Reinoso, Díaz, & Abril, 2017). These results of the FTIR spectra indicted that steam explosion pretreatment did not have a significant effect on the functional groups of the camellia seed oil. This is because the functional groups of the control group and the treatment group showed similar patterns, and the chemical functional groups did not change significantly.

### 3.9 Volatile compounds

Flavor is an important quality criterion for camellia oil. The untreated camellia oil imparted a slight odor with oily and botanical flavor notes, while the steam‐exploded camellia oil presented an attractive nutty and caramel‐like odor. Therefore, identification of flavor compounds is a demand for quality control of camellia oil production. To reveal the difference in flavor notes, HS GC‐MS was conducted to identify the volatile compounds in untreated and steam‐exploded camellia oil. Volatile compounds are considered to be major contributors to the overall flavor characteristics (Zhang, Wang, Yuan, Yang, & Liu, 2016). About 40 volatile compounds were identified and characterized, including hydrocarbons, esters, aldehydes, acids, alcohol, naphthalene, benzene derivatives, and furans (Table 2). The profiles of volatile compounds were changed in steam explosion process. Compared with untreated camellia oil, some new minor volatile compounds were generated, such as p‐xylene, 1,3‐dimethyl‐benzene, esters, and furans. However, the main volatile compounds were still hydrocarbons.

**Table 2:** Volatile compounds identified in untreated and steam‐exploded camellia seed oil (1.6 MPa 30 s)

NO	Compound name	0 MPa	1.6 MPa 30 s
Hydrocarbons			
1	Pentane	Y	Y
2	Methyl‐cyclopentane	Y	Y
3	1,3‐dimethyl cyclopentane	Y	Y
4	n‐hexane	Y	Y
5	2‐methyl‐hexane	Y	Y
6	2,3,4‐trimethyl‐hexane	N	Y
7	Cyclohexane, methyl‐	Y	Y
8	Cyclohexane, ethyl‐	Y	Y
9	1,3‐dimethyl‐cyclohexane	Y	Y
10	1,4‐dimethyl‐cyclohexane	N	Y
11	2‐methyl‐heptane	Y	Y
12	3‐methyl‐heptane	Y	Y
13	Heptane	Y	Y
14	3‐methyl‐hexane	Y	N
15	Octane	Y	N
16	2‐methyl‐octane	N	Y
17	2‐methyl‐nonane	N	Y
18	2,5‐dimethyl‐nonane	N	Y
Esters			
19	9‐octadecenoic acid(Z)‐phenylmethyl.ester	N	Y
20	Formic acid heptyl ester	N	Y
21	Carbonic acid decyl dodecyl ester	N	Y
22	Stearic acid(octadecyloxy)propyl ester	Y	Y
23	N‐Benzyl‐2‐amino cinnamate, methyl ester	N	Y
Aldehydes			
24	2‐heptenal,(Z)	N	Y
25	Heptanal	Y	Y
26	Octanal	Y	N
27	Nonanal	N	Y
Acids			
28	Fumaric acid	N	Y
29	Hexanoic acid	Y	Y
30	Glycocholic acid	Y	Y
Alcohol			
31	1‐heptanal	N	Y
32	2‐propyl‐1‐heptanol	N	Y
33	4‐methyl‐heptanol	Y	N
Naphthalene			
34	2‐methyl‐decalin	N	Y
Benzene derivatives			
35	p‐xylene	N	Y
36	Methylhydroquinone, 2TMS derivative	Y	Y
37	1,3‐dimethyl‐benzene	N	Y
Furans			
38	2‐pentyl‐furan	N	Y
39	2‐furanmethanol	N	Y
40	3‐methyl‐ furan	N	Y

N: not identified as volatile compounds; Y: identified as volatile compounds.

Furan derivatives were a new class of volatile compounds in camellia seed oil after steam explosion. During thermal processing of foods, furans are generated by the Maillard, lipid oxidation/degradation, and caramelization reactions, which contributes to the fruity, sweet, and nutty characteristics of heated foods (Zou, Gao, He, & Yang, 2017). Three furans were qualified as 2‐pentyl‐furan, 2‐furanmethanol, and 3‐methyl‐furan in this study. 2‐furanmethanol (with an earthy, mild sweet and oily odor) was the unique volatile compounds in treated oil, which is mainly formed from pentoses in thermal reactions (Zhang et al., 2018). The presence of 2‐furanmethanol in camellia oil after steam explosion implies that Maillard reaction occurred during steam explosion.

## 4 CONCLUSION

In this study, steam explosion pretreatment was applied to the extraction of camellia seed oil for the first time, and the effect on extraction yield, physicochemical properties composition, oxidative stability, bioactive compounds, ^1^H NMR, and FTIR of camellia seed oil was investigated. Under the optimal conditions (1.6 MPa 30 s), the camellia seed oil reached the highest percent yield (86.56%). Compared with untreated materials, oil extracted by steam explosion exhibited superior physicochemical properties and antioxidant activities. It is worth mentioning that the fatty acid composition of the oil samples was not affected by the steam explosion pretreatments, and the high levels of oleic acid and palmitic acid were preserved. These results were confirmed by the ^**1**^H NMR and FTIR analyses, indicating that the chemical functional groups of the steam‐exploded oil did not change remarkably. Volatile compounds, such as furans in steam‐exploded camellia seed oil, imply that Maillard reaction may occur during steam explosion. In addition, SEM micrographs confirmed that steam explosion pretreatment efficiently promoted the release of oil by breaking down the cellular structure of camellia seed. On balance, steam explosion pretreatment is a promising environmental friendly technology for oil extraction in the food industry.

## CONFLICT OF INTEREST

There are no conflicts of interest to declare.
